# Posttraumatic ventral urethral fistula: a case report

**DOI:** 10.4076/1757-1626-2-8644

**Published:** 2009-09-03

**Authors:** Mehmet Yucel, Sahin Kabay, Levent Sahin, Mustafa Koplay, Soner Yalcinkaya, Tayfun Cucioglu, Namik Kemal Hatipoglu

**Affiliations:** 1 Department of Urology, Dumlupinar University Faculty of Medicine, 43270 Kutahya, Turkey; 2 Department of Anesthesiology, Dumlupinar University Faculty of Medicine, 43270 Kutahya, Turkey; 3 Department of Radiology, Dumlupinar University Faculty of Medicine, 43270 Kutahya, Turkey; 4 Department of Urology, Patnos Goverment Hospital, Afyon, Turkey; 5 Department of Family Medicine, Haydarli Goverment Hospital, Agri, Turkey; 6 Department of Urology, S.B. BagcÄ±lar Education Hospital, Istanbul, Turkey

## Abstract

**Introduction:**

We present the first case reported in the medical literature of a patient with a posttraumatic urethral fistula accompanied by retraction urethral catheter with balloon.

**Case presentation:**

A 69-year-old man was admitted to our hospital with the recurrence urinary tract infection. The patient reports history of urethral trauma, which is retraction urethral catheter with balloon 2 years ago. Cystoscopy and fistulography were performed, and urethrocutaneous fistula was detected. Initial surgical treatment consisted of surgical debridement of fistula tissue, and a urethral catheterization was performed. After 4 weeks of the operation the urethral fistula resolved. In a follow-up period of 24 months no recurrence and no urinary tract infection were occurred.

**Conclusion:**

Self retraction of the urethral catheter with balloon may result with clinically important urethral fistula. A wide range of possible options such as complete excision of the fistula tract and primary closure may be considered for individual cases.

## Introduction

Urethral fistula is a rare entity, which is usually reported to be a result of infectious complications, trauma or surgery [1,2]. In this case report an old patient with ventral urethral fistula after trauma due to the retraction of urethral catheter with balloon has been presented. Retraction of the urethral catheter with balloon is sometimes observed in some urologic patients with altered consciousness. However, currently a severe clinical condition such as urethral fistula after self retraction of the urethral catheter with balloon has not been reported previously.

## Case report

A 69-year-old Turkish Caucasian man was admitted to our urology clinic with recurrence urinary tract infection and chronic intermittent discharge from the ventral side of the penis. He reported that one week before his admission to our clinic he had experienced urinary retention, and was urethral catheterized in another medical center. However, after four days of the urethral catheterization, the patient had withdrawn the urethral catheter himself without deflating the balloon. His medical history included controlled hypertension disease but not diabetes mellitus. Two years before his last presentation to the clinic, he was diagnosed with bladder stone disease, and was treated with diverticulectomy and cystolithotomy. His systemic examination was not significant for any specific disorder. Local examination revealed an orifice of a possible fistula on the ventral side of the penis. His serum glucose level was 93.8 mg/dl, white blood cell count 10.7 × 10^9^/L. Increased leukocyte count and bacteria were observed in the urine analysis. He was admitted to hospital with a possible diagnosis of urethral fistula. In his initial management broad spectrum antibiotics were used to control the urinary sepsis. Bacteriology confirmed a heavy growth of gram negative basil.

**Figure 1:**
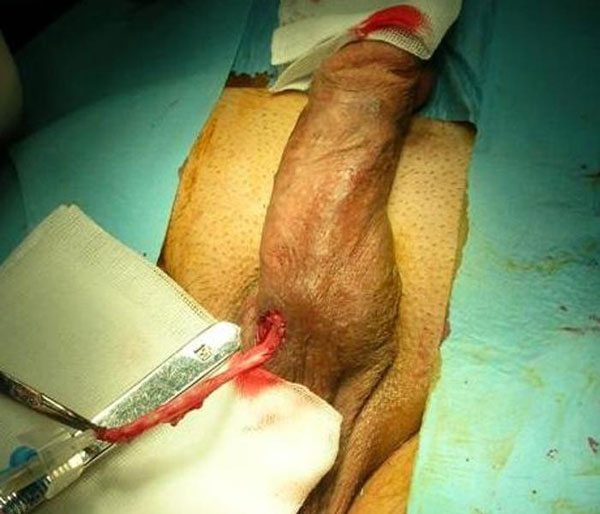
**Urine leakage and urethral fistula tract were observed by the preoperative fistulogram**.

**Figure 2:**
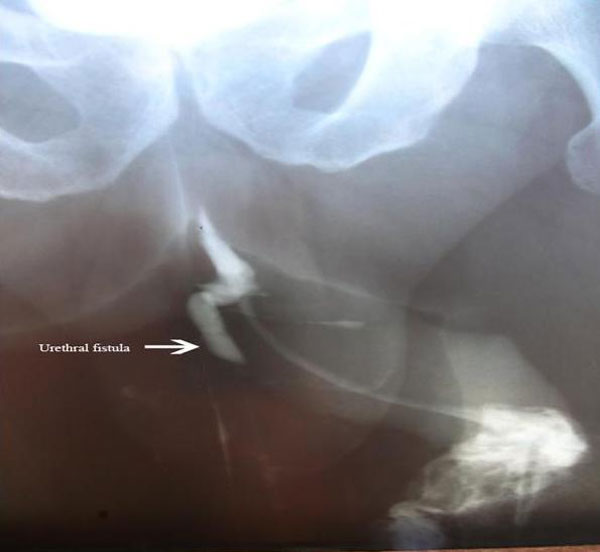
**Intraoperative view: exact exicision of the urethral fistula tract on the ventral side of the urethra**.

**Figure 3:**
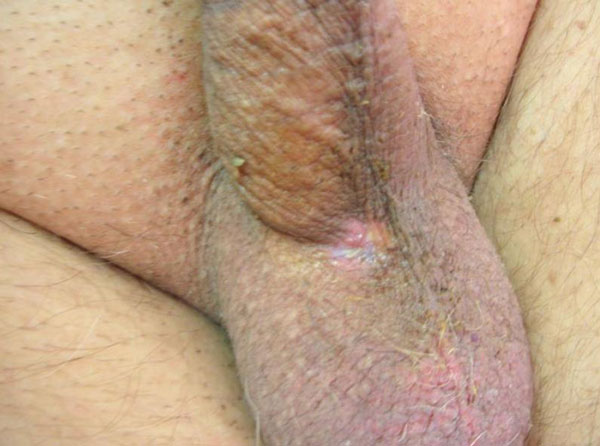
**Postoperative view: excellent wound healing was seen**.

During cystoscopy leakage of the serum physiologic from the orifice on the ventral side of the penis was observed, and no other pathology was noted. A fistulography was performed in order to demonstrate the fistula tract (Figure 1). Total excision of the fistula tract under spinal anesthesia was performed with the intention of definitive treatment of the patient. During the procedure the urethral fistula tract was observed and totally excised (Figure 2). No graft was used during the primary closure after excision of the tract. Urinary diversion was performed with a long-term silastic catheter introduced per urethra. Postoperatively, urine leakage was not observed and the catheter was removed on the third week. Excellent wound healing was seen in figure 3. The patient was able to void without any problems. He remained well with no recurrence of cellulitis or fistula 24 months postoperatively.

## Discussion

Urethral fistulas are classified in two forms such as congenital and acquired. Congenital urethral fistulas are rarely reported anomalies and are generally associated with anorectal malformations. Congenital urethral fistulas are developed either as an embryonic urethral blowout behind a distal congenital obstruction or as an segmental embryonic developmental arrest so that mesoderm fails to encircle the developing groove at the site of the fistula [3,4].

Acquired urethral fistulas are generally reported to be the result of a several clinical conditions such as neoplasms, trauma, or as a complication of infection [3]. Straddle injury and blunt penile trauma are the most known forms of urethral trauma which result with urethral fistulas [5,6]. Complications after some types of penile surgery such as circumcision and corporal-spongial and corporal-saphenous shunts for priapism may also include urethral fistulas [7]-[9]. Also, urethrocutaneous fistula is one of the most frequently observed complications of hypospadias surgery, which requires repeated surgical interventions. Urethral fistula has been reported to be observed in 10% of cases after complex hypospadias repair [10]. Higher urethral fistulas rates have been reported after urethroplasties operations. Al-Qudah H.S. *et al*[11] have reported urethral fistula as a late complication in 18% of cases in their series of 60 patients with urethroplasty for erectile dysfunction-ED, chordee and fistula. However, to our knowledge, no case of a urethral fistula after the retraction of urethral catheter with balloon has been reported previously.

Urethral catheter retraction with balloon may result with the cut of the urethra into parts. Leakage and accumulation of the urine may be caused by diverticulum and urethrocutaneous fistula may be occurred in our patient.

To elucidate the cause of the fistula micturating cystourethrography, cystoscopy, fistulography and magnetic resonance imaging may be considered to identify the fistula tract and the possible coexisting factors contributing to the development of the fistula. In our case fistula tract was identified with fistulography. Cystoscopy was performed in order to document the possible internal fistula orifice in the bladder. To clearly document the pathological condition no further study was needed for our patient.

Some fistulas may be treated with simple procedures such as urethral catheterization and overseeing of fistula [12]. Some more difficult cases merit several different repair techniques and grafts to be considered. In our patient, a complete excision of the fistula tract and simple primary closure procedure was enough to repair the fistula after initial treatment of the urinary sepsis with appropriate antibiotics. He was put on urethral catheterization for three weeks after the surgical intervention.

## Conclusion

In conclusion, self retraction of the urethral catheter with balloon may result with clinically important urethral fistula. A though examination of the patient for the coexisting factors such as diabetes mellitus, diverticulas, stone and/or neoplastic disease is essential in the primary diagnostic workup. A wide range of possible options such as complete excision of the fistula tract and primary closure may be considered for individual cases.

## Consent

Written informed consent was obtained from the patient for publication of this case report and accompanying images. A copy of the written consent is available for review by the Editor-in-Chief of this journal.

## Competing interests

The authors declare that they have no competing interests.

## Authors' contributions

MY, SK and LS have performed the operations and collected data, MK performed the radiological investigation, SY and NKH have written the manuscript. MY and TC review the manuscript.
